# The case management workbook: defining the role of physicians, nurses and case managers

**Published:** 2012-03-12

**Authors:** Wong Loong Mun

**Affiliations:** Chief Care Integration Officer, Care Integration Division, No. 5 Maxwell Road, #10-00 Tower Block, MND Complex, 069110, Singapore Phone: +65 6603 6800 Fax: +65 6820 0728 E-mail: Loong.Mun.WONG@aic.sg

In this slim book of about 100 pages, Murer and her associates try to define the role of each healthcare professional within a multidisciplinary team of care, delineating role, process and responsibility of each member of the team. It rides on the practice of case management as the means to effectively move patients along the continuum of care, particularly, from acute to long-term/community care. It highlights how in the current healthcare environment, it takes a total concerted effort among all to ensure safe and efficient healthcare transition.

The authors provide an administrator’s perspective on how each member of the multidisciplinary team can be aligned with the hospital’s goal of ensuring how a patient can be at the right place at the right time in his/her episode of care. They touch on the financial drivers and incentives of right siting, the obstacles and practical tips in reducing length of stay, the post discharge options (delving into the criteria and financial implications of each post acute care venue), case studies, diagnostic related groups and the importance of managing data. A key consequence in which the authors examine is how an effective case management—if well run—can realize an effective integrated health system where all professionals are aligned with the system.

As an administrator leading teams of care coordinators across acute hospitals in Singapore whose role is to ensure the safe and efficient care transition from hospital to home, the workbook is useful for my teams if it is localized within our healthcare setting. While the audience is written primarily for the American healthcare system whose financial drivers, payment, and range of post acute care services are vastly different from elsewhere, there are ‘jewels’ embedded within where there are ‘boxes’ in each chapter on how to ensure that the goals of case management within a team can be achieved. These boxes tabulate and serve as good pointers or reminders that are generalizable across the practice of case management across healthcare systems.

This book is a workbook and it is written with the intent to give information distilled from complicated jargon and practical tips on how to work together and move the patient along the continuum of care. However, in its attempt to be simple and accessible, it may inadvertently flatten some of the complexities on the ground. For example, in an attempt to flush out the different roles and responsibilities of each member in a team, it may inadvertently have highlighted the physician as not being a team player, behaving in a ‘passive-aggressive’ manner in the management of care. The authors emphasize the utmost importance in the role of the physician as the quarterback and laid out some practical tips on how a case manager and/or nurse can use to work with the physician. This is laudable but again, fails to highlight how the role of the patient and family can complicate the process of discharge.

Strangely, in a book about team-based care, the patient’s voice seems to be glaringly absent. Within our local context, the voices of the patient and caregiver are critical in determining discharge and the post acute care. Very often, for the patient and caregiver, issues of affordability, supportive care at home, end of life care and family dynamics are important key decision factors affecting discharge. If I have to contextualize a similar book for our setting, it is necessary to list the role of the patient/caregiver, obstacles encountered when working with them, and provide them with the information on the options available for post acute care. While the complexities related to patient care are often related to the clinical and functional aspects of care, very often, the case managers are bogged with the social and family aspects of care that are not within the control of the team. The challenge for many case managers is how to weave in the patient and caregiver, collaborate with them to manage care while abiding by the exigencies and drivers of the system. This is a tussle that the case managers in Singapore are often confronted with.

The Case Management Workload is indeed a workbook that will help many who are new to team-based care and the work to integrate care across the continuum. It inspires us to imagine a similar book within our local setting to help in our own little ways the road to an integrated healthcare system.

